# Sub-millikelvin-resolved superconducting nanowire single-photon detector operates with sub-pW infrared radiation power

**DOI:** 10.1093/nsr/nwae319

**Published:** 2024-09-14

**Authors:** Qi Chen, Fei Zhou, Chen Wei, Yue Dai, Haiyong Gan, Labao Zhang, Hao Wang, Hang Yuan, Haochen Li, Jingrou Tan, Guojin Feng, Xuecou Tu, Xiaoqing Jia, Qingyuan Zhao, Lin Kang, Jian Chen, Peiheng Wu

**Affiliations:** Research Institute of Superconductor Electronics, School of Electronic Science and Engineering, Nanjing University, Nanjing 210023, China; Research Institute of Superconductor Electronics, School of Electronic Science and Engineering, Nanjing University, Nanjing 210023, China; Research Institute of Superconductor Electronics, School of Electronic Science and Engineering, Nanjing University, Nanjing 210023, China; Research Institute of Superconductor Electronics, School of Electronic Science and Engineering, Nanjing University, Nanjing 210023, China; Institute of Optics and Laser Metrology Science, National Institute of Metrology, Beijing 100029, China; Research Institute of Superconductor Electronics, School of Electronic Science and Engineering, Nanjing University, Nanjing 210023, China; Hefei National Laboratory, Hefei 230088, China; Research Institute of Superconductor Electronics, School of Electronic Science and Engineering, Nanjing University, Nanjing 210023, China; Hefei National Laboratory, Hefei 230088, China; Research Institute of Superconductor Electronics, School of Electronic Science and Engineering, Nanjing University, Nanjing 210023, China; Research Institute of Superconductor Electronics, School of Electronic Science and Engineering, Nanjing University, Nanjing 210023, China; Research Institute of Superconductor Electronics, School of Electronic Science and Engineering, Nanjing University, Nanjing 210023, China; Institute of Optics and Laser Metrology Science, National Institute of Metrology, Beijing 100029, China; Research Institute of Superconductor Electronics, School of Electronic Science and Engineering, Nanjing University, Nanjing 210023, China; Research Institute of Superconductor Electronics, School of Electronic Science and Engineering, Nanjing University, Nanjing 210023, China; Hefei National Laboratory, Hefei 230088, China; Research Institute of Superconductor Electronics, School of Electronic Science and Engineering, Nanjing University, Nanjing 210023, China; Research Institute of Superconductor Electronics, School of Electronic Science and Engineering, Nanjing University, Nanjing 210023, China; Hefei National Laboratory, Hefei 230088, China; Research Institute of Superconductor Electronics, School of Electronic Science and Engineering, Nanjing University, Nanjing 210023, China; Research Institute of Superconductor Electronics, School of Electronic Science and Engineering, Nanjing University, Nanjing 210023, China

**Keywords:** NETD, infrared detector, SNSPD, photon-counting, thermal imaging

## Abstract

The noise equivalent temperature difference (NETD) indicates the minimum temperature difference resolvable by using an infrared detector. The lower the NETD, the better the sensor can register small temperature differences. In this work, we proposed a strategy to achieve a high temperature resolution using a superconducting nanowire single-photon detector (SNSPD) with ultra-high sensitivity. We deduced the model for calculating the NETD of a photon-counting-type detector and applied it to our SNSPD-based set-up. Experimentally, we obtained an NETD as low as 0.65 mK, which is limited by the background radiation of the environment, and the required infrared radiation power is calculated to be <1 pW. Furthermore, the intrinsic NETD of this SNSPD is estimated to be <0.1 mK. This work demonstrated a sub-mK temperature resolution when using the SNSPD, paving the way for future remote infrared thermal imaging with high temperature resolution.

## Teaser

Superconducting nanowire single-photon detectors improve the thermal detection resolution to sub-millikelvin with sub-pW infrared radiation power.

## INTRODUCTION

Non-contact and sensitive probing of temperature variations is a significant challenge for a wide range of modern applications with weak thermal radiation. For example, direct observation of local energy dissipation in integrated circuits [1–3] and quantum devices [4] has proven to be a hotspot and high temperature resolution is the premise of microscale thermal management in addition to high spatial resolution. Furthermore, remote sensing, such as sensitive monitoring of temperature changes in the ocean, is critical for providing elaborate observations to improve climate models [5] and the resolution of temperature detection in ocean and geological changes needs to be ∼1 mK [6], which requires an infrared detector with high sensitivity and high temperature resolution. A key indicator for evaluating the temperature resolution of an infrared detector is the noise equivalent temperature difference (NETD), which is defined as the minimum temperature difference of the measured target at which the infrared detector can identify.

Traditional infrared detectors can be classified into two types: thermal detectors and photon detectors. Thermal detectors include thermopiles [7], microbolometers [8,9] and thermomechanical detectors [10,11]. A thermal detector converts the thermal effect caused by infrared radiation into electrical signals (such as resistance, current and capacitance) or mechanical stress signals during operation. The greater the intensity of the radiation, the stronger the signals. Thermal detectors can work at room temperature but the NETD can reach tens of mK. For example, Chen *et al*. developed a microbolometer with a SiN*_x_* core material and obtained an NETD of ∼33 mK through integration with metamaterials [12]. Wu *et al*. reported a VO_2_-based microbolometer with an NETD of 64.5 mK [9]. In recent years, studies have shown that thermomechanical detectors can achieve higher temperature resolution. For example, a shape memory polymer resonator achieved a minimum NETD of 6 mK due to its high temperature coefficient of resonance frequency characteristics [10]. Das *et al*. reported a subwavelength perforated detector that can achieve a minimum NETD of 4.5 mK by converting infrared radiation into the phase shift of interference fringes [11].

Photon detectors may have a higher response speed and sensitivity than thermal detectors. They use the basic principle of generating photogenerated carriers that further form photocurrents to achieve infrared detection. Traditional infrared photon detectors include InSb detectors [13], mercury cadmium telluride (MCT) detectors [14], quantum well infrared (QWIP) photodetectors [15] and type-II superlattice detectors [16]; the NETDs are distributed between 20 and 100 mK. In addition, photon detectors based on other materials are being gradually developed. Tang *et al*. reported HgTe colloidal quantum dot photodetectors, which could achieve an NETD of 14 mK [17]. Yakunin *et al*. accomplished thermal imaging by using a low-dimensional perovskite-like tin halide material and achieved a temperature resolution of ∼13 mK [18]. Ding *et al*. developed an integrated up-conversion detector by using the temperature-dependent photoluminescence of semiconductor materials such as GaAs and finally obtained an NETD of tens of mK [19].

The general model of the NETD is shown in Equation (1):


(1) \begin{eqnarray*}
\textit{NETD} = \frac{{{\mathrm{\Delta }}T}}{{{\mathrm{\Delta }}SNR}} = \frac{{{\mathrm{\Delta }}T}}{{{\mathrm{\Delta }}{{V}_{\rm s}}/{{V}_{\rm n}}}},
\end{eqnarray*}

where Δ*T* represents the temperature difference change in the target (or the source), Δ*SNR* defines the relative signal-to-noise ratio of the infrared detector, and Δ*V*_s_ and *V*_n_ represent the variation in the output signal and the root mean square of the noise in the infrared detector, respectively. Different infrared detectors do not have the same noise sources, which include background radiation noise, carrier generation-recombination noise, thermal noise and electronic noise. Studies have shown that the ultimate limit in terms of the performance of a photodetector is when all sources of noise are negligible compared with background noise—a condition known as background-limited infrared photodetection [20]. Thus, the signal fluctuation is dominated by Poisson noise, whose intensity is equal to the square root of the background response. The high temperature resolution of the detector depends on the high signal-to-noise ratio, which further depends on the high sensitivity. Therefore, an efficient strategy for achieving ultralow NETD is to improve the sensitivity of the detector.

High sensitivity is a prerequisite for the detector to obtain high temperature resolution in applications with weak thermal radiation. The dominant noise of traditional infrared detectors comes from the target radiation in some thermal imaging applications (with a high radiation power) and the NETD is independent of the sensitivity. However, once the thermal radiation generated by the target is very weak, the noise of traditional infrared detectors may exceed the intrinsic fluctuation of thermal radiation, degrading performance due to limited sensitivity. Therefore, the exploration of infrared detectors with higher sensitivity to achieve high-resolution thermal detection and imaging has become a topic worthy of in-depth research.

Single-photon detectors can detect a minute energy packet of light. Therefore, it is possible to use a single-photon detector with higher sensitivity to obtain a higher resolution of temperature than when using traditional infrared detectors. Superconducting nanowire single-photon detectors (SNSPDs) have been applied in long-distance lidar [21], quantum key distribution [22] and deep space laser communication [23]. The detection principle can be described as follows: a photon is absorbed by a superconducting nanowire, which destroys a great number of Cooper pairs and causes a superconducting phase transition, resulting in measurable resistance in the nanowire; finally, an electrical pulse is output with the assistance of a bias current *I*_B_. In a high-performance SNSPD, if the current increases to a certain value that is lower than the superconducting critical current, then a further increase in the bias current will no longer increase the quantum efficiency of the detector. Then, the SNSPD is considered to have a saturated quantum efficiency [24]. The ultralow superconducting energy gap (<5 meV) enables the SNSPD to achieve highly sensitive detection of lower-energy photons [25,26]. In recent years, the detection wavelength of the SNSPD has gradually expanded from the visible/near-infrared to the mid- and long-wave infrared regions. In 2023, Taylor *et al*. adjusted the component ratio of the superconducting film to lower the detection threshold and increased the detection cut-off wavelength to 29 μm [27]. Hampel *et al*. developed a mid-infrared single-photon imager with 64 pixels from a single-pixel SNSPD [28]. Nanjing University has previously developed SNSPDs with detection wavelengths ranging from 1 to 10 μm [29,30].

This work makes full use of the low-noise and high-sensitivity performance of SNSPDs to propose a high-temperature-resolution detection technology. The equation for calculating the NETD with a photon-counting-type detector was developed from the basic definition. Furthermore, we demonstrated that the NETD could reach a sub-mK level, while an infrared radiation power of <1 pW was needed based on the SNSPD and the theoretical model.

### Temperature resolution model

**Figure 1.**
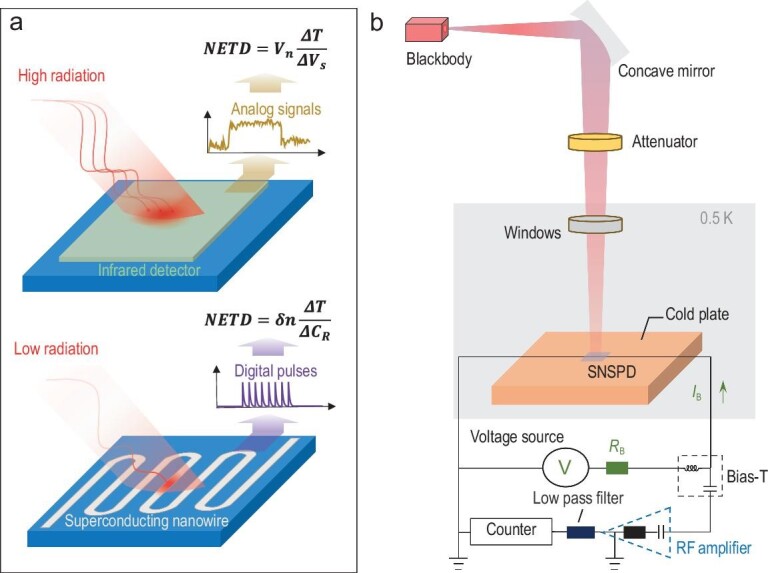
(a) Temperature-resolving principle of detectors with different operating modes (traditional infrared detector and SNSPD). (b) Schematic diagram of the measurement system.

The equation for calculating the NETD with a photon-counting-type detector is deduced based on the SNSPD first. A schematic of the difference between the traditional infrared detector and the SNSPD photon-counting detector is shown in Fig. 1a. The SNSPD works in photon-counting mode and infrared photons can be converted into discrete digital electrical pulses by superconducting nanowires. The rise and fall times of electrical pulses are usually within hundreds of picoseconds and tens of nanoseconds, respectively, allowing a very high photon count to be obtained. The SNR model can be expressed as follows from the perspective of the detector:


(2) \begin{eqnarray*}
SNR = \frac{{{{C}_R}}}{{\delta n}},
\end{eqnarray*}

where *C*_R_ represents the photon count rate, which varies with the temperature of the source. δ*n* represents the root mean square of the background count rate *B*_c_, which is independent of the temperature of the source. When the source temperature changes Δ*T* (Δ*T* is a small value), the variation in *C*_R_ can be expressed as Δ*C*_R_, so the relative SNR will be Δ*C*_R_/δ*n*. Combined with Equation (1), the calculation model of the corresponding NETD is obtained via:


(3) \begin{eqnarray*}
\textit{NETD} = {\mathrm{\ }}\frac{{{\mathrm{\Delta }}T}}{{{\mathrm{\Delta }}{{C}_R}/\delta n}}.
\end{eqnarray*}

Furthermore, the Allan deviation is introduced by considering the effect of the integration time *τ* on the NETD and the time domain characteristics of δ*n* can be calculated as follows:


(4) \begin{eqnarray*}
\delta n\!\left( \tau \right) = {\mathrm{\ }}{{\left[ {\frac{1}{{2\left( {M - 1} \right)}}\mathop \sum \limits_{j = 1}^{M - 1} {{{( {{{X}_{j + 1}} - {{X}_j}} )}}^2}} \right]}^{0.5}},
\end{eqnarray*}

where *τ* = *mτ*_0_ and *τ*_0_ represents the unit time. Both *M* and *X*_j_ represent the background counts and the average value of the background count rate within *τ*, respectively. An increase in *τ* can effectively reduce δ*n* and subsequently reduce the NETD according to the Allan deviation.

There are three sources of *B*_c_, which are the count rate *N*_r_ caused by the background radiation (in this experiment, the dark counts caused by cosmic rays and radioactivity have not been considered because the SNSPD was installed in the shielded dilution refrigerator in the laboratory and the dark counts caused by cosmic rays and radioactivity are negligible. In the future, when the SNSPD is applied for space observation, cosmic rays and radioactivity are inevitable sources of dark counts, which can be shielded by using special designs, such as magnetic shielding, aluminum shielding and multilayered shielding [31,32]), the intrinsic dark count rate *N*_d_ of the SNSPD and the count rate *N*_e_ caused by the circuit. Experimental studies have shown that *N*_r_ obeys a negative binomial distribution, which can be taken as a Poisson distribution when the mode of the photons is complicated. Therefore, the probability of *N*_r_ is *P*(*N*_r_) = *N^N^*^r^⋅*e*^−^*^N^*/*N*_r_!, where *N* represents the mean value of *N*_r_ [33]. Therefore, the root mean square of *N*_r_ can be directly obtained as *N*^0.5^. The main factors affecting *N*_d_ include structural defects, current and detector operating temperature. The *N*_d_ can be effectively suppressed by optimizing the superconducting film growth process and nanofabrication. *N*_e_ is less affected by the current and is generally negligible compared with the first two sources at a high bias current. As previously mentioned, if *N*_r_ is the main source of noise, then the detection performance of the SNSPD can be limited by shot noise. Therefore, δ*n* = *N*^0.5^ and obeys the time law δ*n*/*τ*^0.5^. The *τ*-dependent NETD can be obtained via:


(5) \begin{eqnarray*}
\textit{NETD}\!\left( \tau \right) = \frac{{{\mathrm{\Delta }}T}}{{{\mathrm{\Delta }}{{C}_R}/\delta n}}{{\left( {\frac{1}{\tau }} \right)}^{0.5}}.
\end{eqnarray*}

The NETD decreases with increasing *τ*. For example, if *τ* increases by 100 times, then the NETD can be reduced by 10 times.

### Measurement set-up

The measurement system is shown in Fig. 1b. A Mikron M305 blackbody source with 0.995 emissivity and an aperture with a width of 24.4 mm was used. The operation temperature *T*_b_ of the blackbody source can be adjusted to between 400 and 1000 K with a resolution of 0.1 K. The infrared radiation generated by the blackbody source passes through the reflector and the adjustable neutral density attenuator, and then enters the dilution refrigerator. There are four windows in the dilution refrigerator; the material used for the outermost window and the innermost window is CaF_2_ and the material used for the remaining two windows is ZnSe. The temperatures of each window from the outside to the inside are 300, 40, 3 and 0.5 K, respectively. After passing through all the windows, the infrared radiation is eventually coupled to the detection area of the SNSPD. To attenuate the infrared signal to the single-photon level, the defocusing of the optical path is deliberately adjusted. Since the spot of the infrared radiation is larger than that of the active area, the density of infrared photons on the active area can be considered to be the same.

**Figure 2.**
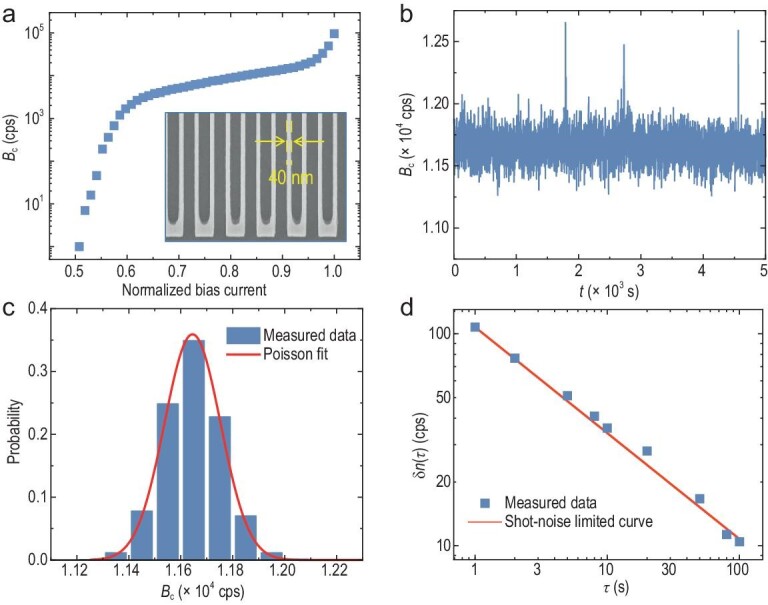
Noise analysis of the SNSPD. (a) Normalized bias current-dependent background count rate *B*_c_ of the SNSPD; the inset shows an SEM image of the 40-nm-wide nanowires. (b) Time domain stability and (c) statistics of *B*_c_ when the normalized bias current is set to 0.9; the red curve in (c) shows the Poisson distribution. (d) Relation between δ*n* and the integration time; the red curve shows the theoretical level of the shot-noise limitation.

This work also calculated the actual absorbed infrared radiation power *P*_a_ by the SNSPD and concluded that *P*_a_ is ∼0.1–1 pW under different *T*_b_ conditions. This result is much lower than the infrared radiation power required by conventional temperature-resolving detectors [9]. The ‘Absorbed infrared radiation power calculation’ section at the end of the paper is used for detailed analysis. In the experiments, the SNSPD was installed on the cold stage in which the temperature was maintained at 0.5 K. A Keysight 2400 DC voltage source meter with a series resistance *R*_B_ (*R*_B_ = 100 kΩ) was used to provide a stable and adjustable bias current *I*_B_. *I*_B_ is input into the SNSPD chip through the DC terminal of the bias-T. The inset in Fig. 2a shows a scanning electron microscopy (SEM) image of nanowires of the fabricated SNSPD. To enhance the responsivity of the detector, the width of the nanowires was reduced to 40 nm. When the SNSPD detects photons and undergoes a superconducting phase transition, the electric pulses are output to an external room-temperature counting circuit through the radio frequency port of the bias-T. To improve the SNR of the response pulse when the counter is used, a room-temperature amplifier with a gain of ≤63 dB was adopted. Furthermore, a lowpass filter with a cut-off frequency of 22 MHz is added to the front end of the counter.

## RESULTS AND ANALYSIS

The background count rate *B*_c_ is increased by the normalized bias current (the bias current *I*_B_ divided by the critical current *I*_SW_, *I*_B_/*I*_SW_), as shown in Fig. 2a, where the blackbody source was shuttered and the outermost window of the refrigerator was blocked. There is a gentle zone with a low increasing rate at 0.6–0.9 *I*_B_/*I*_SW_, which is different from the intrinsic dark counts of the SNSPD, indicating that the source of *B*_c_ is dominated by background radiation from the measurement system. A sharp increase in *B*_c_ occurs when *I*_B_ is close to 1, where *B*_c_ is dominated by the intrinsic dark counts of the SNSPD. Therefore, a reasonable *I*_B_/*I*_SW_ of 0.9 is chosen for further evaluation of the time domain stability and the statistical characteristics of *B*_c_, as shown in Fig. 2b and c. *B*_c_ exhibits a homogenous fluctuation and the corresponding mean value remains stable over a long detection time (5000 seconds). Furthermore, the statistical characteristics of *B*_c_ obeyed the Poisson distribution, as shown by the curve in Fig. 2c. This indicates that the SNSPD detection results reveal the quantum photon statistics of a background thermal light source. The mean value of *B*_c_ is 1.2 × 10^4^ cps (counts per second) and the root mean square of δ*n* is 108 cps. In addition, δ*n* under different integration times *τ* is obtained by calculating the Allan deviation, as shown in Fig. 2d. δ*n* always coincides with the shot-noise-limited level at different *τ* values. For example, δ*n* can decrease to 34 cps when *τ* increases to 10 s. Thus, when the SNSPD detects infrared radiation, the total noise is dominated by *B*_c_.

**Figure 3.**
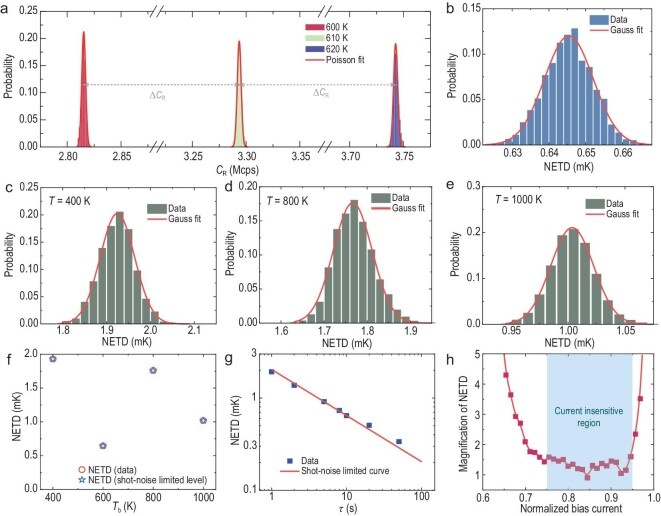
NETD results for the SNSPD data set. (a) Distributions of *C*_R_ when *T*_b_ is set to 600, 610 and 620 K. The curve defines the Poisson distribution. (b) Distribution of the NETD under a 10-s integration time. (c) NETD at *T*_b_ = 400 K. (d) NETD at *T*_b_ = 800 K. (e) NETD at *T*_b_ = 1000 K. Curves in (c)–(e) show Gaussian fits. (f) Comparison of the experimental data and theoretical shot-noise-limited levels of NETD at different *T*_b_. (g) Variation in NETDs versus the integration time (*T*_b_ = 600 K) and the curve defines shot-noise-limited detection. (h) Relative NETD versus the normalized bias current and the curve guides the eyes.

The optical response of the SNSPD to the blackbody source was measured. First, the temperature *T*_b_ of the blackbody source was controlled to be *T*_b_ = 600 K. The optical path and *I*_B_/*I*_SW_ = 0.9 were kept unchanged. The detection time was increased to collect thousands of *C*_R_ counts for the blackbody sources at 600 and 610 K, respectively. With the gradual increase in *t*, the direct *C*_R_ results fluctuated at low frequencies and did not exceed 0.1 Hz. Please refer to the ‘C_R_ with an fast fourier transform (FFT) filter’ section in the Supporting Information for detailed explanations. These fluctuations are independent of the SNSPD itself and can be eliminated by using a high-pass FFT filter (Figs S1 and S2). Figure 3a shows the mean value of *C*_R_ and its distribution at different *T*_b_. The statistical characteristics of *C*_R_ also obey the Poisson distribution, revealing the quantum photon statistics of the blackbody source by virtue of the low noise and high sensitivity of the SNSPD. The measured *C*_R_ values are 2.82 and 3.29 Mcps at *T*_b_ = 600 and 610 K, respectively. Therefore, the increase in *C*_R_ (Δ*C*_R_) caused by the temperature difference in the blackbody source at 10 K is 0.47 Mcps. Furthermore, *T*_b_ was increased to 620 K and the corresponding *C*_R_ distribution was measured under the same integration time. The calculated Δ*C*_R_ resulting from the increase in *T*_b_ from 610 to 620 K is 0.45 Mcps, which is approximately equal to the previous result (where *T*_b_ increases from 600 to 610 K). The NETD of the SNSPD is calculated to be 0.65 mK at *τ* =10 s by using Equation (5), as shown in Fig. 3b. The NETDs that were measured at different times are independent of each other, so we fitted the results by using a Gaussian function, as shown by the curve in Fig. 3b. Furthermore, we adjusted *T*_b_ to lower and high values (*T*_b_ = 400, 800 and 1000 K) and measured the corresponding *C*_R_ under an integration time of 10 s (Fig. S3). The NETDs at these blackbody temperatures were further calculated, as shown in Fig. 3c–e. The measured NETDs were 1.93, 1.76 and 1.02 mK for temperatures of 400, 800 and 1000 K, respectively. Notably, the attenuation was changed at different source temperatures. Thus, these NETDs did not reveal the intrinsic performance of this SNSPD. Fortunately, all these values were as low as their shot-noise-limited detection, as shown in Fig. 3f. Furthermore, the *τ*-dependence of the NETD is also discussed. The NETD variation can be obtained by changing *τ* while keeping *T*_b_ = 600 K, as shown in Fig. 3g. The curve in Fig. 3g represents the shot-noise limitation. The experimental results showed that the measured NETD was consistent with the shot-noise limitation at different integration times.

Figure 3h shows the variation in the relative NETD (the current-dependent NETD divided by the minimal value) versus the normalized bias current, *I*_B_/*I*_SW_. The NETD monotonically decreases with increasing *I*_B_ in the range of *I*_B_/*I*_SW_ < 0.75. In this range, the NETD is mainly limited by the sensitivity of the SNSPD and its *C*_R_. As *I*_B_ increases, the NETD reaches a relatively low value because of the relatively high sensitivity and *C*_R_ of the SNSPD. Furthermore, the NETD increases rapidly with increasing *I*_B_ in the range of *I*_B_/*I*_SW_ > 0.95 due to the high intrinsic dark count. Thus, the NETD can be maintained at a low level, with *I*_B_/*I*_SW_ ranging from 0.75 to 0.95. This region can be referred to as the current insensitive region because the NETD is less affected by fluctuations in the current.

**Figure 4.**
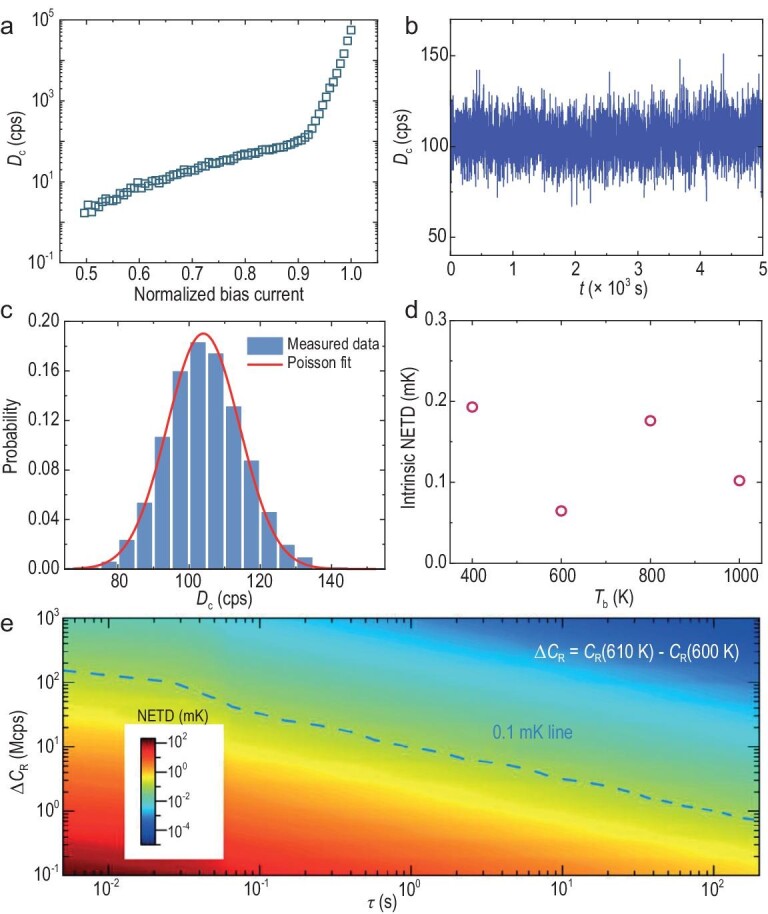
(a) Current-dependent dark count rate *D*_c_ of the SNSPD. (b) Time domain stability of *D*_c_. (c) Statistics of *D*_c_; the curve shows the Poisson distribution. (d) Intrinsic NETDs of the SNSPD. (e) Possible improvement in the NETD in the future according to Equation (5) and Fig. 3b.

To analyse the intrinsic NETD of these SNSPDs, the current-dependent dark count rate *D*_c_ of the SNSPD with a 0.5 K shielding cover was measured (Fig. 4a). In the SNSPD, the width of the superconducting nanowire was reduced to 40 nm to improve the detection responsivity at mid-infrared wavelengths. Therefore, the measurement of the intrinsic *D*_c_ shows a tail below nearly 0.9 of the critical current in the curve, as in Ref. [34], which is slightly different from the curves of traditional SNSPDs [35] due to size-effect-controlled mechanisms [36].

It can be clearly seen that *B*_c_ is always much larger than *D*_c_ (e.g. at *I*_B_/*I*_SW_ = 0.9). Figure 4b shows that *D*_c_ is time-independent. Furthermore, the statistics show that *D*_c_ still obeys the Poisson distribution at a high bias current because the dark counts are stochastic independent events and have ultralow probabilities within the detection time window (approximately tens of nanoseconds) of the SNSPD. Thus, the fluctuation can be calculated as 10.2 cps, which is 10 times lower than δ*n* obtained under the same bias current (Fig. 4c). For potential applications, such as remote sensing, the background can be avoided by using cold optics technology. Thus, the intrinsic NETD can only consider the dark count rate *D*_c_ of the SNSPD. As shown in Fig. 4d, the intrinsic NETD of the SNSPD can be estimated to be lower than 0.1 mK.

We extrapolate that this approach can further improve the NETD, as shown in Fig. 4e. Based on the experimental results in Fig. 3b, we estimated the NETD by increasing Δ*C*_R_ and *τ* according to Equation (5). Benefitting from the low dark count rate of the SNSPD, an increase in the integration time *τ* is feasible for further improvements in the temperature resolution. Another route, namely an increase in Δ*C*_R_, requires improvement in the maximum photon count rate of the SNSPD. First, improvement in the system detection efficiency and the development of an SNSPD array are two efficient technical solutions for increasing *C*_R_ and Δ*C*_R_. The system detection efficiency consists of the quantum efficiency, the absorption efficiency and the coupling efficiency. Low-gap superconducting films are helpful for obtaining a saturated quantum efficiency. The design and fabrication of wide-spectrum, high-absorption and polarization-insensitive optical cavities can improve the absorption efficiency. The construction of low-temperature space optical systems or the enlargement of the active area can improve the coupling efficiency. An SNSPD array can overcome the contradiction between the active area and the kinetic inductance, which is significant for improving the photon count rate. For the SNSPD, both Δ*C*_R_ and *τ* are related to the NETD: NETD ∼ Δ*C*_R_/*τ*^0.5^. Thus, *τ* can be greatly reduced if the photon count rate is large enough. For example, if the blackbody source temperature *T*_b_ = 600 K and the sub-mK resolution capability remains unchanged, then, if the photon count rate increases by 100-fold (from ∼10^6^ to ∼10^8^ cps), the integration time *τ* can be reduced from 10 s to 1 ms. Second, the intrinsic dark count *N*_d_ can be reduced by reducing δ*n* through the development of amorphous superconducting films to reduce material defects, optimize the fabrication process of superconducting nanowires to reduce structural defects, optimize the structural layout of nanowires to suppress current crowding and signal reflection, and reduce the operating temperature and the thermal-excited phase slip ratio of the SNSPD. Thus, Fig. 4e is a guide for the selection of a reasonable option for achieving an NETD of <0.1 mK with a high photon count rate and a low integration time or with a long integration time and a low photon count rate in future practical applications. For example, with the advantage of high sensitivity in long-wave infrared, the SNSPD will also have the potential to conduct direct high-resolution thermal detection and imaging for cold targets (such as quantum systems with weak thermal radiation). An appropriate increase in the integration time or the development of an SNSPD array with a large active area to improve the photon count rate (reduce the integration time) is beneficial to reveal the energy dissipation mechanisms in quantum matter. This will be an interesting research topic in the future.

## CONCLUSION

In summary, this work takes advantage of the SNSPD, such as high sensitivity and low noise, to explore the potential in thermal detection and imaging with a high temperature resolution. An NETD model based on photon-counting detection was developed. It has been demonstrated that the NETD can break through to sub-mK and reach the level of shot-noise limitation, verifying the intrinsic performance of high temperature resolution. Moreover, the infrared radiation power that was absorbed by the SNSPD was estimated to be <1 pW. Improvements in the absorption efficiency and photon count rate and a decrease in the dark count rate could further optimize the temperature resolution and integration time.

## METHODS

### Materials and detector fabrication

The preparation procedures for the SNSPD are as follows.

First, a 5-nm-thick polycrystalline NbN superconducting film was grown on a Si substrate (with a 270-nm-thick SiO_2_ layer on the surface) by using a DC magnetron sputtering system at room temperature (23°C). The Nb target had a high purity of 99.999%. The substrate was cleaned with Ar ions for 15 s before film deposition and the beam current and pressure were set at 300 mA and 0.5 mTorr, respectively. The vacuum in the main chamber was pumped to 10^−6^ Pa before N_2_ and Ar were passed through. During the sputtering process, the mixed reaction gas had an Ar/N_2_ ratio of 7:1, a pressure of 2 mTorr, a current of 1.05 A and an electric power of 452 W. The thickness of the NbN film depended on the deposition rate (1 nm/s) and time.

Second, the Au electrode was prepared on the surface of the NbN thin film by using ultra-violet lithography and a DC magnetron sputtering system; the XR-1541–006 hydrogen silsesquioxane electron beam etchant resist was spin-coated and baked (the bake temperature and time were 90°C and 4 min, respectively); and the meandering nanowire pattern was subsequently prepared by using the electron beam lithography. The width of the nanowire was 40 nm, the spacing between adjacent nanowires was 100 nm and the entire active area was 10 μm × 10 μm. The sample was developed by using a ZX-238 developer at 20°C for 4 min and then baked at 90°C for 2 min to further cure the e-beam resist after exposure; the nanowire pattern was subsequently transferred to the NbN film by using the reactive ion etching technique. The etch gas and the ratio were SF_6_/CHF_3_ and 40/20 sccm, respectively. The etch pressure, electric power and time were set to 4 Pa, 80 W and 35 s, respectively. The resistance–temperature (*R*–*T*) curve of the SNSPD was measured by using the four-terminal method, so both the superconducting transition temperature *T*_c_ = 5.29 K and the corresponding transition width Δ*T* = 1.33 K were obtained (Fig. S4).

### Absorbed infrared radiation power calculation

In this study, the infrared radiation power *P*_a_ absorbed by the SNSPD was calculated during the experiment. Fig. S5a shows the procedure in which the infrared radiation from the blackbody source passes through the neutral density attenuator with adjustable attenuation and through all windows of the dilution refrigerator. Finally, it arrives at the active area of the SNSPD. The size of the blackbody source is much less than the distance *r* between the blackbody source and the SNSPD. The centers of the blackbody source and the SNSPD active area lie on the same optical axis. The atmospheric transmittance is 1, the area of the blackbody source is *S*, the attenuation of the neutral density attenuator is *O*_d_ (*O*_d_ = lg(*P*_in_/*P*_out_), where *P*_in_ and *P*_out_ represent the incident and transmitted infrared radiation power, respectively), the total spectral transmittance of the dilution refrigerator window is *T*_r_, the active area of the SNSPD is *A*_d_ and the spectral absorption efficiency of the nanowires is *η*_0_. The infrared radiation power *P*_0_ incident on the active area can be calculated within the source spectral band (*λ*_1_ ≤ *λ* ≤ *λ*_2_) via:


(6) \begin{eqnarray*}
{{P}_0} = \mathop \int \nolimits_{{{\lambda }_1}}^{{{\lambda }_2}} {{L}_\lambda }S{\mathrm{\Omega }}{\rm d}\lambda = \mathop \int \nolimits_{{{\lambda }_1}}^{{{\lambda }_2}} {{L}_\lambda }{{A}_d}{\mathrm{\Omega }}D{{T}_r}{{\eta }_0}{\rm d}\lambda ,
\end{eqnarray*}

where *λ*_1_ and *λ*_2_ represent the upper and lower limits of the transmission spectrum, respectively, and *L*_λ_ represents the wavelength-dependent blackbody spectral monochromatic radiance. *L*_λ_ can be expressed in the following form from Planck's law:


(7) \begin{eqnarray*}
{{L}_\lambda } = \frac{{\varepsilon\! \left( \lambda \right){{c}_1}}}{{\pi {{\lambda }^5}\Big[ {{{e}^{\frac{{{{c}_2}}}{{\lambda {{T}_b}}}}} - 1} \Big]}},
\end{eqnarray*}

where *ε*(λ) represents the emissivity of the blackbody source; *ε*(λ) ≡ 1 represents a perfect blackbody; *c*_1_ and *c*_2_ represent Planck's first and second radiation constants, respectively; *c*_1_ = 2π*hc*^2^ = 3.7415 × 10^4^ W⋅μm^4^⋅cm^−2^; *c*_2_ = *hc*/*k* = 1.43 879 × 10^4^ μm⋅K; and *T*_b_ is the operating temperature of the blackbody source. The unit of *L*_λ_ is W⋅μm^−1^⋅cm^−2^. The radiation solid angle *Ω* of the detector to the blackbody source is:


(8) \begin{eqnarray*}
\Omega = \frac{{{{A}_d}}}{{{{r}^2}}}.
\end{eqnarray*}

We measured *r* = 0.75 m. Fig. S5b shows the total spectral transmittance of the dilution refrigerator windows and the absorption spectrum of the meander NbN nanowires. The total spectral transmittance is ∼40% in the transmission band of 1–10 μm. FDTD Solutions simulation software was used to calculate the absorption spectrum. The plane wave incidence mode was set during the simulation process to obtain the absorption efficiencies for the transverse electric and transverse magnetic waves. The total absorption efficiency is half of the sum of the absorption efficiencies for the TE and TM waves, which is shown as a red curve in Fig. S5b. This work mainly studied the intrinsic photon detection performance of superconducting nanowires and did not integrate a high-efficiency infrared cavity. Therefore, the average absorption efficiency of the nanowires in the band of 1–10 μm is ∼10%.

We further calculated the actual absorbed infrared power *P*_a_ by the detector based on the above parameters and the results are shown by the blue points in Fig. S5c. For example, when *T*_b_ = 600 K, the attenuation of the neutral density attenuator was set to *O*_d_ = 1 to prevent the latching-up of the detector and *P*_a_ was calculated to be <1 pW. We adjusted the attenuation to obtain a *P*_a_ of between 0.1 and 1 pW if *T*_b_ was changed. The higher sensitivity of the SNSPD makes the *P*_a_ in this work much lower, providing a new idea for remote high-resolution thermal imaging scenarios. In particular, the NETD of the SNSPD always decreases with the integration time *τ* as the square root of *τ* due to the white-noise-dominated mechanism. The *τ* of the SNSPD can be decreased to milliseconds or even less if the performance of the SNSPD is improved while maintaining the mK or sub-mK temperature resolution.

## Supplementary Material

nwae319_Supplemental_File

## References

[1] Tessier G, Bardoux M, Filloy C et al. High resolution thermal imaging inside integrated circuits. Sensor Review 2007; 27: 291–7. 10.1108/02602280710821425

[2] Mecklenburg M, Hubbard WA, White ER et al. Nanoscale temperature mapping in operating microelectronic devices. Science 2015; 347: 629–32. 10.1126/science.aaa2433 25657242

[3] Xue H, Qian R, Lu W et al. Direct observation of hot-electron-enhanced thermoelectric effects in silicon nanodevices. Nat Commun 2023; 14: 3731. 10.1038/s41467-023-39489-z 37349328 PMC10287675

[4] Halbertal D, Cuppens J, Shalom MB et al. Nanoscale thermal imaging of dissipation in quantum systems. Nature 2016; 539: 407–10. 10.1038/nature19843 27786173

[5] Caesar L, Rahmstorf S, Robinson A et al. Observed fingerprint of a weakening Atlantic Ocean overturning circulation. Nature 2018; 556: 191–6. 10.1038/s41586-018-0006-5 29643485

[6] Riser SC, Freeland HJ, Roemmich D et al. Fifteen years of ocean observations with the global Argo array. Nat Clim Change 2016; 6: 145–53. 10.1038/nclimate2872

[7] Hsu AL, Herring PK, Gabor NM et al. Graphene-based thermopile for thermal imaging applications. Nano Lett 2015; 15: 7211–6. 10.1021/acs.nanolett.5b01755 26468687

[8] Chen C, Li C, Min S et al. Ultrafast silicon nanomembrane microbolometer for long-wavelength infrared light detection. Nano Lett 2021; 21: 8385–92. 10.1021/acs.nanolett.1c02972 34606292

[9] Wu B, Zhang Z, Chen B et al. One-step rolling fabrication of VO_2_ tubular bolometers with polarization-sensitive and omnidirectional detection. Sci Adv 2023; 9: eadi7805. 10.1126/sciadv.adi7805 37851806 PMC10584336

[10] Adiyan U, Larsen T, Zárate JJ et al. Shape memory polymer resonators as highly sensitive uncooled infrared detectors. Nat Commun 2019; 10: 4518. 10.1038/s41467-019-12550-6 31586068 PMC6778134

[11] Das A, Mah ML, Hunt J et al. Thermodynamically limited uncooled infrared detector using an ultra-low mass perforated subwavelength absorber. Optica 2023; 10: 1018–28. 10.1364/OPTICA.489761

[12] Chen C, Huang Y, Wu K et al. Polarization insensitive, metamaterial absorber-enhanced long-wave infrared detector. Opt Express 2020; 28: 28843–57. 10.1364/OE.403105 33114794

[13] High-Speed MWIR Camera for Range & Science Applications: FLIR RS8500 . https://flir.netx.net/file/asset/26960/original (19 August 2024, date last accessed).

[14] Varavin VS, Sabinina IV, Sidorov GY et al. Photodiodes based on p-on-n junctions formed in MBE-grown n-type MCT absorber layers for the spectral region 8 to 11 μm. Infrared Phys Technol 2020; 105: 103182. 10.1016/j.infrared.2019.103182

[15] Palaferri D, Todorov Y, Bigioli A et al. Room-temperature nine-µm-wavelength photodetectors and GHz-frequency heterodyne receivers. Nature 2018; 556: 85–8. 10.1038/nature25790 29579743

[16] Lee H-J, Jang A, Kim YH et al. Comparative advantages of a type-II superlattice barrier over an AlGaSb barrier for enhanced performance of InAs/GaSb LWIR nBn photodetectors. Opt Lett 2021; 46: 3877–80. 10.1364/OL.435479 34388764

[17] Tang X, Ackerman MM, Guyot-Sionnest P. Thermal imaging with Plasmon resonance enhanced HgTe colloidal quantum dot photovoltaic devices. ACS Nano 2018; 12: 7362–70. 10.1021/acsnano.8b03871 29985583

[18] Yakunin S, Benin BM, Shynkarenko Y et al. High-resolution remote thermometry and thermography using luminescent low-dimensional tin-halide perovskites. Nat Mater 2019; 18: 846–52. 10.1038/s41563-019-0416-2 31263225

[19] Ding H, Lv G, Cai X et al. An optoelectronic thermometer based on microscale infrared-to-visible conversion devices. Light 2022; 11: 130. 10.1038/s41377-022-00825-5 PMC9079085 35525849

[20] Bianconi S, Mohseni H. Recent advances in infrared imagers: toward thermodynamic and quantum limits of photon sensitivity. Rep Prog Phys 2020; 83: 044101. 10.1088/1361-6633/ab72e5 32018242 PMC7282310

[21] Zhang B, Guan Y-Q, Xia L et al. An all-day lidar for detecting soft targets over 100 km based on superconducting nanowire single-photon detectors. Supercond Sci Technol 2021; 34: 034005. 10.1088/1361-6668/abd576

[22] Li W, Zhang L, Tan H et al. High-rate quantum key distribution exceeding 110 mb s^–1^. Nat Photonics 2023; 17: 416–21. 10.1038/s41566-023-01166-4

[23] Hao H, Zhao Q-Y, Huang Y-H et al. A compact multi-pixel superconducting nanowire single-photon detector array supporting gigabit space-to-ground communications. Light 2024; 13: 25. 10.1038/s41377-023-01374-1 PMC10803749 38253520

[24] Engel A, Renema JJ, Il'in K et al. Detection mechanism of superconducting nanowire single-photon detectors. Supercond Sci Technol 2015; 28: 114003. 10.1088/0953-2048/28/11/114003

[25] Korneev A, Kouminov P, Matvienko V et al. Sensitivity and gigahertz counting performance of NbN superconducting single-photon detectors. Appl Phys Lett 2004; 84: 5338–40. 10.1063/1.1764600

[26] Holzman I, Ivry Y. Superconducting nanowires for single-photon detection: progress, challenges, and opportunities. Adv Quantum Technol 2019; 2: 1800058. 10.1002/qute.201800058

[27] Taylor GG, Walter AB, Korzh B et al. Low-noise single-photon counting superconducting nanowire detectors at infrared wavelengths up to 29 µm. Optica 2023; 10: 1672–8. 10.1364/OPTICA.509337

[28] Hampel B, Mirin RP, Nam SW et al. A 64-pixel mid-infrared single-photon imager based on superconducting nanowire detectors. Appl Phys Lett 2024; 124: 042602. 10.1063/5.0178931 PMC11070947 38711922

[29] Chen Q, Ge R, Zhang L et al. Mid-infrared single photon detector with superconductor Mo_0.8_Si_0.2_ nanowire. Sci Bull 2021; 66: 965–8. 10.1016/j.scib.2021.02.024 36654252

[30] Chen Q, Dai Y, Li F et al. Design and fabrication of superconducting single-photon detector operating in 5–10 μm wavelength band. Acta Phys Sin 2022; 71: 248502. 10.7498/aps.71.20221594

[31] Barthel J, Sarigul-Klijn N. A review of radiation shielding needs and concepts for space voyages beyond Earth's magnetic influence. Prog Aerosp Sci 2019; 110: 100553. 10.1016/j.paerosci.2019.100553

[32] Gohel A, Makwana R. Multi-layered shielding materials for high energy space radiation. Radiat Phys Chem 2022; 197: 110131. 10.1016/j.radphyschem.2022.110131

[33] Cheng R, Zhou Y, Wang S et al. A 100-pixel photon-number-resolving detector unveiling photon statistics. Nat Photonics 2023; 17: 112–9. 10.1038/s41566-022-01119-3

[34] Marsili F, Bellei F, Najafi F et al. Efficient single photon detection from 500 nm to 5 μm wavelength. Nano Lett 2012; 12: 4799–804. 10.1021/nl302245n 22889386

[35] Chiles J, Charaev I, Lasenby R et al. New constraints on dark photon dark matter with superconducting nanowire detectors in an optical haloscope. Phys Rev Lett 2022; 128: 231802. 10.1103/PhysRevLett.128.231802 35749181

[36] Bartolf H, Engel A, Schilling A et al. Current-assisted thermally activated flux liberation in ultrathin nanopatterned NbN superconducting meander structures. Phys Rev B 2010; 81: 024502. 10.1103/PhysRevB.81.024502

