# Supplementary material for: Sub-millikelvin-resolved superconducting nanowire single-photon detector operates with sub-pW infrared radiation power
Source: Natl Sci Rev. 2024 Sep 14;12(1):nwae319. doi: 10.1093/nsr/nwae319 (PMC11737322; doi:10.1093/nsr/nwae319)
Supplement: nwae319_Supplemental_File [file nwae319_supplemental_file.docx]

**Sub-millikelvin-resolved superconducting nanowire single-photon detector operates with sub-pW infrared radiation power: *Supporting Information***

Qi Chen^1,†^, Fei Zhou^1,†^, Chen Wei^1,†^, Yue Dai^1,†^, Haiyong Gan^2,*^, Labao Zhang^1,3,*^, Hao Wang^1,3^, Hang Yuan^1^, Haochen Li^1^, Jingrou Tan^1^, Guojin Feng^2^, Xuecou Tu^1^, Xiaoqing Jia^1,3^, Qingyuan Zhao^1^, Lin Kang^1,3^, Jian Chen^1^ and Peiheng Wu^1^

^1^Research Institute of Superconductor Electronics, Nanjing University, Nanjing 210023, China

^2^National Institute of Metrology, Beijing 100029, China

^3^Hefei National Laboratory, Hefei 230088, China

^*^Correspondence: Haiyong Gan (ganhaiyong@nim.ac.cn), and Labao Zhang (Lzhang@nju.edu.cn).

^†^These authors contributed equally to this work.

## 1. *C*_R_ with an FFT filter

**Fig. 1** shows the time-dependent *C*_R_. The temperature of the blackbody is *T*_b_ = 1000 K, the normalized biased current is 0.9, the attenuation *O*_d_ of the neutral density attenuator is *O*_d_ = 2, the integration time for one count event is *τ* = 1 s, and the total detection time is *t* = 2000 s. The blue line shows the measured data, which have a low-frequency fluctuation, and the frequency is no larger than 0.1 Hz. Considering the intrinsic properties of superconducting detectors, it has been shown that the fluctuations in the bias current applied to the SNSPD remain below 1 nA for a long time, which is very small compared to the superconducting transition current (2.1 μA) and does not lead to significant fluctuations in the superconductivity. Therefore, the stable superconductivity renders the optical response characteristics of the SNSPD independent of the detection time *t*.

In this work, the sources of these low-frequency fluctuations are analyzed from two main perspectives. First, the blackbody source itself will produce a certain temperature drift under long-term working conditions. Second, the background environment may also experience a slow temperature fluctuation during the measurement process. Both factors cause the number of radiated photons to fluctuate.

For experimental comparison and verification, a wideband thermal power probe S401C produced by Thorlabs was adopted (the resolution was 1 µW, the measurement uncertainty at a wavelength of 1064 nm was ±0.3%, and the response time was 1.1 s) when keeping *T*_b_ = 1000 K unchanged. The thermal power probe was installed at the front end of the blackbody source at a 0.35 m distance. The center of the entry pupil stop of the thermal power probe lies on the same optical axis as the center of the blackbody source, and the entire optical path is protected by a special metal sleeve to isolate the stray photons. In addition, a narrowband filter with a central wavelength of 1064 nm and a passband half-height and width of 3 nm (with a transmittance > 90%) was installed on the front optical path of the thermal power probe. The output signal of the thermal power probe was connected to the digital optical power and energy meter, and the infrared radiation power density read out by the probe was collected by the automatic acquisition software for a long time. The results are shown by the blue curve in **Fig. 2**. The direct measurement results show that the power density of the blackbody source does have low-frequency fluctuations in the time domain, which confirms the previous analysis, indicating that the low-frequency fluctuations in the *C*_R_ are mainly caused by the blackbody source. Future experiments with more stable blackbody sources could fundamentally eliminate the fluctuation effect. After spectrum analysis of the infrared radiation power density, the effect of low-frequency fluctuations can be effectively eliminated by adopting high-pass filtering with a cutoff frequency of 0.1 Hz. The filtered results are also shown by the red curve in **Fig. 2**.

Therefore, we adopted the fast Fourier transform (FFT) high-pass filtering technology to filter out the low-frequency fluctuations in the *C*_R_ to eliminate the influence of the external environment on the detection results and determine the intrinsic photon detection capability of the SNSPD. The filtering results are shown by the red curve in **Fig. 1**. The cutoff frequency is set to 0.1 Hz; that is, noise with a fluctuation time scale exceeding 10 s is filtered out. Finally, we can obtain stable *C*_R_ results through FFT high-pass filtering technology.





**Supplementary Figure 1.** Time-dependent *C*_R_. The blue and red lines show the measured data and the results obtained after high-pass FFT filtering (with a cutoff frequency of 0.1 Hz), respectively.


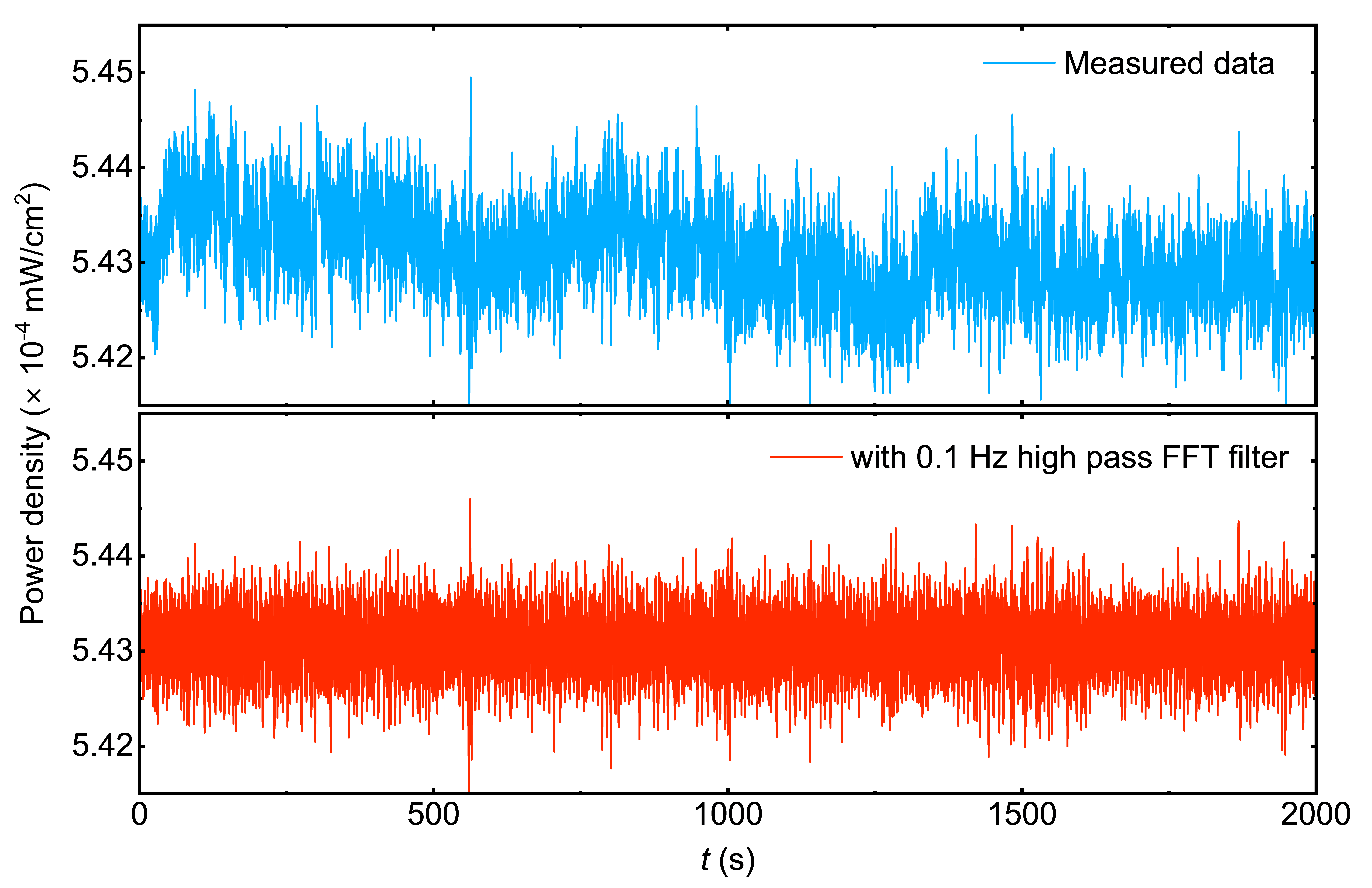


**Supplementary Figure 2.** Time-dependent infrared radiation power density readout obtained by a thermal power probe. The blue and red lines show the measured data and the results obtained after high-pass FFT filtering (with a cutoff frequency of 0.1 Hz), respectively.

The *C*_R_ distributions of the SNSPD at different blackbody temperatures are shown in **Fig. 3**a-c. The total detection time was *t* = 2000 s, the standard temperature *T*_b_ of the blackbody source was set to 400, 800, and 1000 K, respectively, and the temperature difference was Δ*T* = 5 or 10 K. To prevent the latch-up of the detector effectively, the attenuation *O*_d_ of the neutral density attenuator was adjusted properly for different *T*_b_. If *T*_b_ = 800 or 1000 K, then *O*_d_ = 2; if *T*_b_ = 400 K, then *O*_d_ = 0 (indicating no attenuation). The temperature difference Δ*T* will cause an obvious difference in the *C*_R_, and there will be a linear relationship between the *C*_R_ and *T*_b_.


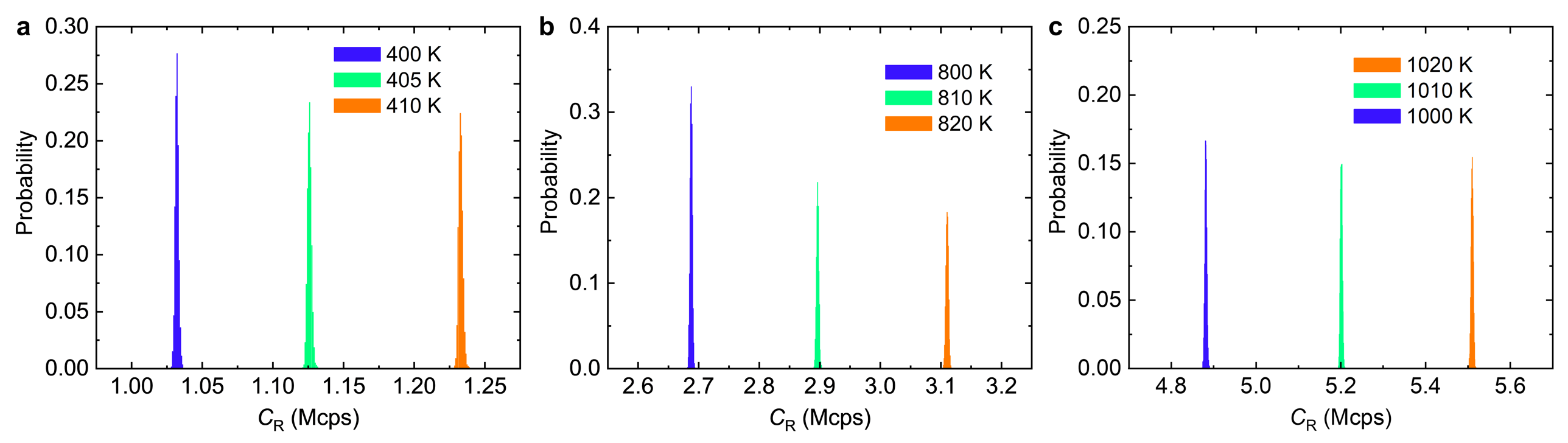


**Supplementary Figure 3.** The distributions of *C*_R_ at different *T*_b_.

## 2. Resistance-temperature curve of nanowires


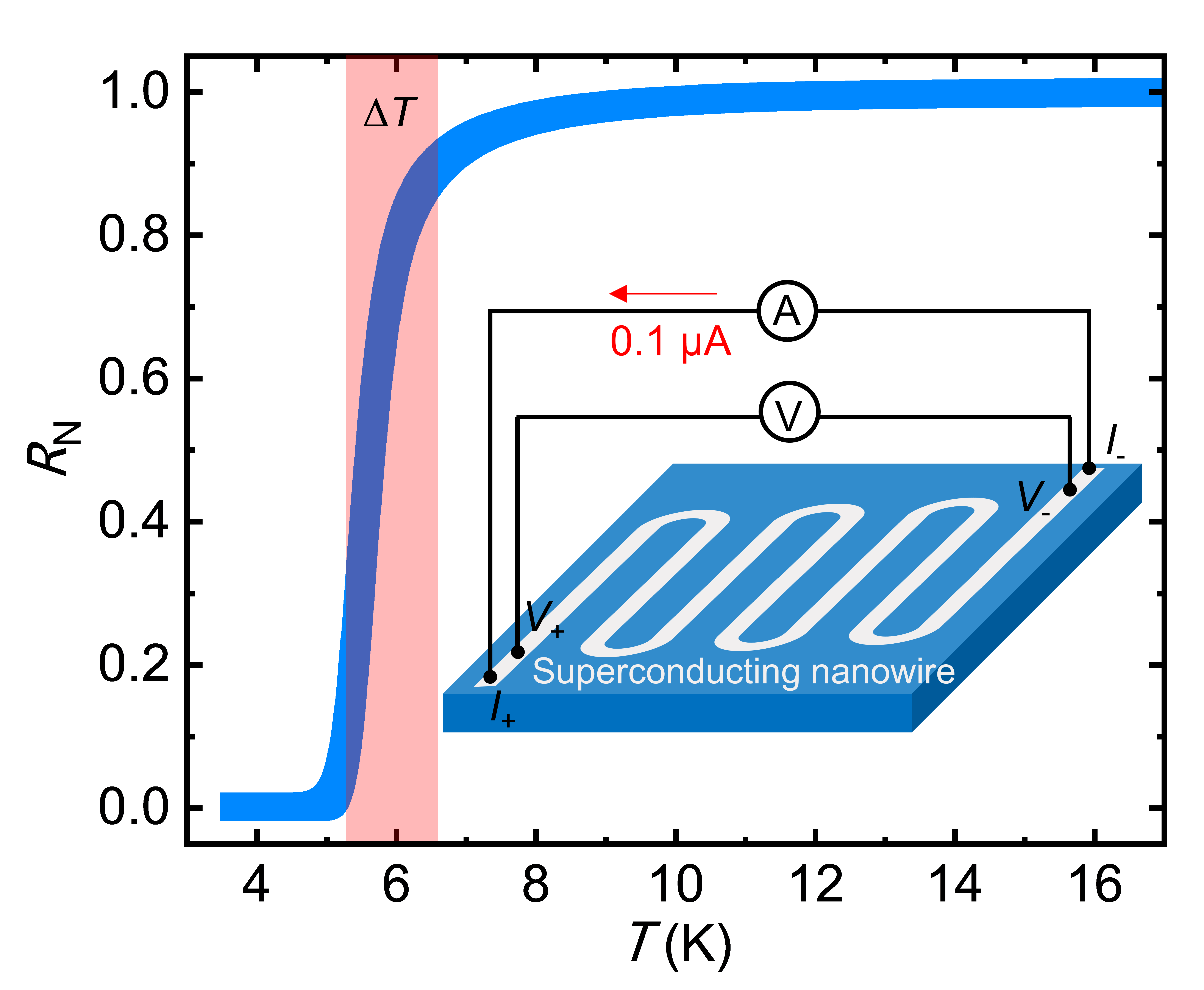


**Supplementary Figure 4.** The resistance-temperature (*R*-*T*) curve measured by the four-terminal method. The bias current was 0.1 μA, much lower than the critical current.

## 3. Absorption of infrared radiation power





**Supplementary Figure 5.** Calculation of the required absorbed infrared radiation power *P*_a_. **a.** Schematic diagram of the transmission process of infrared radiation from the blackbody source to the detector; **b.** The spectral transmittance curves of the dilution refrigerator and the spectral absorption efficiency of nanowires; **c.** The values of *P*_a_ at different blackbody temperatures; the blue data points show the results in the experiments.
